# A meta-analysis of exosome in the treatment of spinal cord injury

**DOI:** 10.1515/med-2021-0304

**Published:** 2021-07-15

**Authors:** Hanxiao Yi, Yang Wang

**Affiliations:** Department of Oncology, Sun Yat-Sen Memorial Hospital, Sun Yat-Sen University, No. 107, YanJiang Road, Haizhu District, Guangzhou, GuangDong Province, 510000, China; Department of Spine Surgery, Third Affiliated Hospital of Sun Yat-Sen University, Sun Yat-Sen University, Guangzhou, GuangDong Province, 510000, China

**Keywords:** exosome, rodent animals, acute spinal cord injury, locomotor function recovery, meta-analysis

## Abstract

**Context:**

There are no recommended therapeutic agents for acute spinal cord injury (SCI) due to the pathophysiological complexity of the injury.

**Objective:**

The objective of this study is to investigate the efficacy of various exosomes and potential factors impacting the efficacy of exosomes.

**Methods:**

We searched the PubMed, EMBASE, Web of Science, Medline, Scopus, and Cochrane Library databases to systematically collect articles comparing the locomotor function of SCI rodents undergoing exosome treatment and untreated SCI rodents. No language was preferred.

**Results:**

Pooled analysis revealed that the locomotor function recovery of SCI rodents receiving exosomes was greatly improved (583 rats, 3.12, 95% CI: 2.56–3.67, *p* < 0.01; 116 mice, 2.46, 95% CI: 1.20–3.72, *p* < 0.01) compared to those of control rodents. The trial sequential analysis demonstrated the findings of the meta-analysis with the cumulative *Z*-curve crossing the upper monitoring boundary for the benefit and reaching the adjusted required information size. However, the origin of the exosome, SCI model, and administration method determined the therapeutic effect to some extent.

**Conclusions:**

Despite the proven therapeutic effects of exosomes on SCI rodents, the results should be interpreted cautiously considering the diversity *in vivo* and *in vitro* in relation to future trials.

## 1 Introduction

Spinal cord injury (SCI), a life-threatening disorder, is closely associated with deficits in locomotor function and sensation [1] and has an annual prevalence of 10–83 cases per million [2], with 90% of these cases being traumatic SCI. Early decompression is usually recommended for patients with SCI; however, postsurgical drug treatment strategies are still lacking.

Secondary inflammation after SCI directly induces extension of the injury, which is the result of ischemia, inflammation, secretion of excitotoxic substances, and worsening deficits in locomotor function and sensation resulting from oxidative stress [3]. Due to the absence of therapeutic agents, rat and mouse models of SCI (induced by ischemia, compression, contusion, and transection) are often used in the laboratory to develop innovative therapies. Melatonin [4], high-dose methylprednisolone [5], a Rho inhibitor [6], and riluzole [7] are currently being tested in humans and animals, but the efficacy of these agents is still the subject of debate. Therapeutic effects are often observed in laboratory animals but not in humans, which suggests that the specific functional mechanism of a drug rather than the drug itself is important. Usually, in addition to being efficacious in humans, drugs should have limited side effects and acceptable costs. Additionally, some agents remain in the animal experiment stage of development. In this context, an increasing number of novel drugs for SCI are emerging from the laboratory.

Many studies have shown that mesenchymal stem cells (MSCs) are promising cell therapy agents for both humans and animals with SCI, possibly through inhibition of inflammatory cascades [8,9,10]. As the product of stem cells, exosomes are considered to be important paracrine modulators and also the next generation of cell-free therapeutic agents for humans with SCI [11,12]. Exosomes are nano-sized 20- to 150-nm-diameter particles composed of a lipid bilayer that wraps RNA, DNA, and soluble proteins [13,14]. Due to their lipid bilayers, exosomes freely move through the blood, are absorbed by target cells, and can even pass through the blood–brain barrier.

Despite the unlikelihood of complete recovery, more researchers are acknowledging that exosomes can provide satisfactory improvements in motor function for exosomes. To determine whether exosomes are neuroprotective in rodent models of SCI, a systematic review of the efficacy of exosomes for the treatment of SCI is needed. Thus, we performed a systematic review and meta-analysis of data from studies investigating rodent models of SCI to assess the efficacy of exosomes for acute traumatic SCI.

## 2 Methods

### 2.1 Search strategies

This meta-analysis was limited to published articles on rodents and was performed by searching PubMed, EMBASE, Web of Science, Medline, Scopus, and the Cochrane Library databases (from inception to 2021). The search strategy is as follows: ((exosomes[title/abstract]) OR (extracellular vesicles[title/abstract]) OR (nano-sized vesicles[title/abstract]) OR (micro-vesicles)) AND (SCI[title/abstract]). The reference lists of the included articles were also searched to identify other studies. To perform a comprehensive search, we did not limit the “species”; articles reporting an unexpected “species” were excluded from the study selection process. A detailed database search strategy is provided in Table S1.

### 2.2 Study selection

All studies were stored as bibliographic references in NoteExpress (Aegean Sea Software Company, Beijing, China) and selected by two independent researchers (YW and XWL) based on the inclusion criteria. After primary selection, all articles were downloaded, and the articles that did not meet the inclusion criteria were excluded by browsing the specific content. A debate was resolved in consultation with a third investigator (HXY).

### 2.3 Eligibility criteria

The processing of articles followed the **PICOS** principle.

Type of participants (**P**): All studies included laboratory rats and mice subjected to acute SCI. Studies using nonmechanical methods such as radiation, electricity, and biochemical substances were excluded from the analysis.

Type of intervention (**I**): Studies that compared exosome administration to PBS, saline, or culture supernatant administration were included regardless of administration frequency, administration mode, and origin of the exosome.

Type of control (**C**): Studies with at least two intervention arms, with animals in the control group receiving placebo and animals in the experimental group receiving exosome administration, were included in this analysis.

Type of outcome (**O**): Studies that evaluated the locomotor function of the hind limbs of rats with the Basso, Beattie & Bresnahan (BBB) scale rather than the Basso Mouse Scale (BMS) and those that evaluated the locomotor function of the hind limbs of mice with the BMS rather than the BBB scale were included.

Type of study (**S**): All studies assessing the locomotor function recovery of SCI mice and rats were included.

### 2.4 Data extraction and quality assessment

Two skilled researchers (YW and XWL) independently extracted data from all articles meeting the inclusion criteria. The following data were extracted from the included studies: author, year, species, weight, the damaged segment of the spinal cord, anesthetic, SCI model, origin of exosomes, dose, administration frequency, and administration mode. When the data were presented as figures rather than tables, GetData Graph Digitizer 2.25 (Fedorov) was used to obtain the data. Based on our observations, the first analysis of mice and rats was usually conducted within 48 h, which may explain why the scores were presented as 0; in such cases, this measurement was not considered the first measurement. The quality of all included studies was evaluated by SYRCLE’s tool.

### 2.5 Outcome measurements

Behavioral improvement was assessed and recorded using the BBB locomotor rating scale for hind limb motor function in rats. The BBB scale, which ranges from 0 (no hind limb movement) to 21 (normal locomotion), was used to analyze specific improvements in locomotor function. The BMS, which ranges from 0 (no hind limb movement) to 9 (normal locomotion), was also used to assess motor function in mice. The movements of the hip, knee, and ankle joints were recorded when animals were allowed to move freely in an open field for 5 min.

### 2.6 Statistical analysis

The data from all included studies were summarized and analyzed by using R software version 3.6.3 (University of Auckland, New Zealand) and meta-package. All results reported in this review are presented as standardized mean differences (SMDs) with 95% CIs for outcomes. A random-effect model was used to analyze the data when heterogeneity was significant (*p ≤* 0.05 or *I* ^2^ > 50%); otherwise, a fixed-effect model was used. Publication bias was tested by Egger’s *t*-test with R software version 3.6.3 and is presented as a funnel plot. Subgroup analyses of different models of SCI, administration modes, and measurement time points were also conducted. Trial sequential analysis (TSA) was conducted by using TSA software.

## 3 Results

The studies included in this meta-analysis were reported according to the Preferred Reporting Items for Systematic Review and Meta-Analysis (PRISMA) (Table S2) [15].

### 3.1 Article selection process

**Figure 1:**
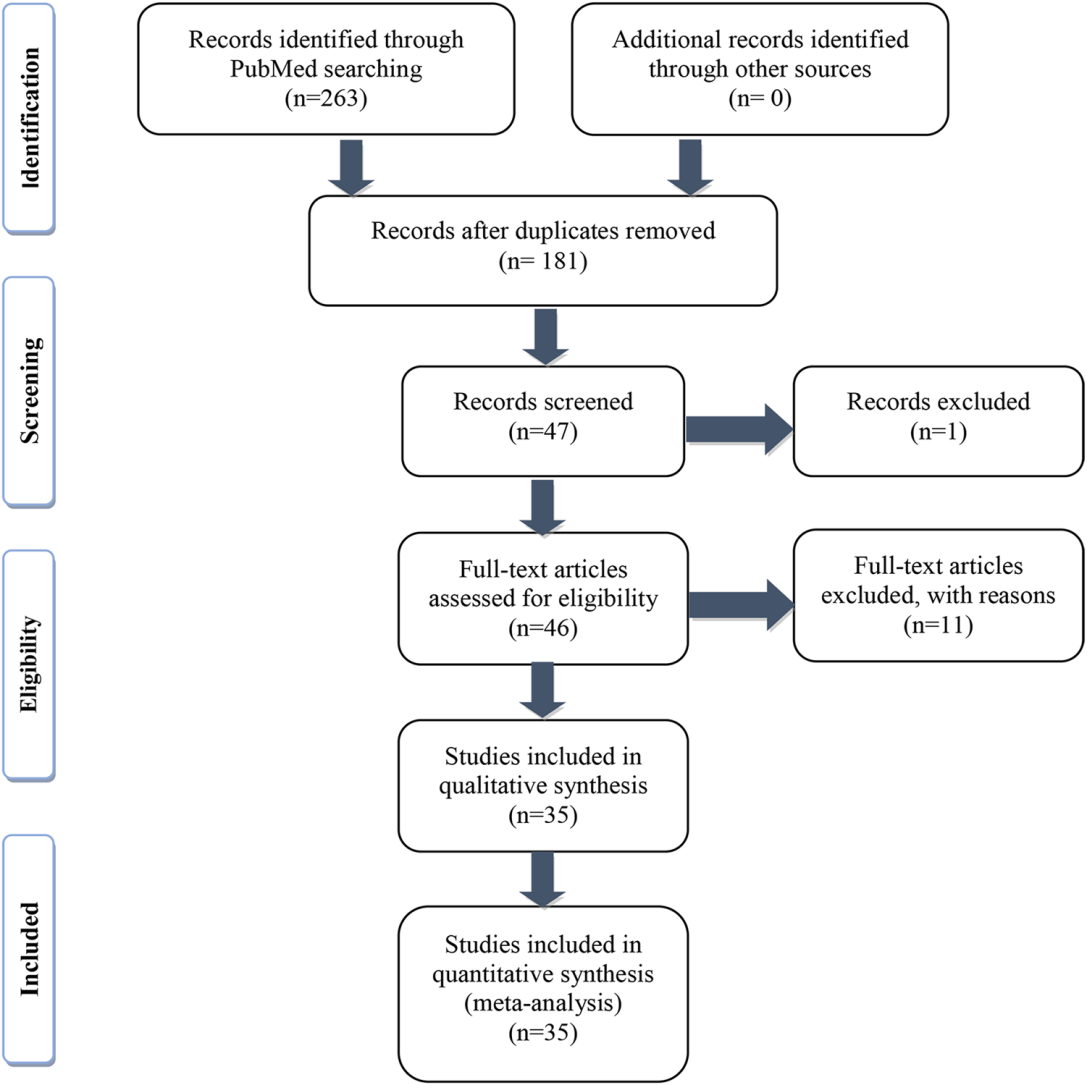
Summary of the article selection process.

The article selection process is shown in Figure 1. A total of 263 unique titles were retrieved from the databases. After removing duplicates and browsing the abstracts, 47 articles entered the full-text screening process. In this process, 1 article was excluded due to a lack of access. Following the full-text screening process, 11 studies, including one study that was withdrawn for plagiarism, two studies that used a different rating scale, one that utilized a rabbit model of SCI, and seven other studies that aim to investigate the pathophysiological development of SCI rats, were excluded. Ultimately, 35 articles, including two articles published in Chinese and 33 published in English, fully met the inclusion criteria set by the researchers.

### 3.2 Study characteristics

As we investigated improvements in the motor function of both rats and mice, data for the two species were collected separately (Table 1). To collect as many critical factors as possible, the sample size of each group was not considered but is provided in the following figures and tables. Functional improvements in rats were reported in 29 studies, whereas the remaining six studies reported improved outcomes in mice.

**Table 1:** Characteristics of included studies reporting rats and mice

Author	Year	Species	Gender	Weight	Segment	Anesthesia	Model	Origin of exosome	Dose	Timing of injection	Administration mode
**Rat**											
Huang	2017	SD rats	Male	180–220 g	T10	10% chloral hydrate (0.3 mL/kg)	Strike, 8 g × 40 mm	BMSC	100 μg	30 min PI	Tail vein injection
Pei	2017	NA	NA	NA	T10	10% chloral hydrate (0.33 mL/kg)	Strike, 200 kilodyne	BMSC	50 μL (200 μg/mL)	1 h PI	Tail vein injection
Ruppert	2017	SD rats	Male	225–250 g	T10	1.5 L/min of 2–3% isoflurane	Strike, 50 kdynes with 1 s dwell	hUC-MSC	10^9^ particles/mL (1 mL)	3 h PI	Tail vein injection
Kang	2018	SD rats	NA	180–220 g	T9/10	10% chloral hydrate (0.33 mL/kg)	Strike,10 g × 25 mm	miR-21, or PTEN siRNA transfected BMSC	NA	NA	Tail vein injection
Huang	2018	SD rats	NA	180–220 g	T10	10% chloral hydrate (3 mg/kg)	Strike, 8 g × 40 mm	HUVEC, miR-126 transfected HUVEC	100 μg	30 min PI	Tail vein injection
Jia	2018	SD rats	Male	200–250 g	T10	4% isoflurane and 2% isoflurane	Strike, 200 kilodyne	BMSC	200 μL (200 μg/mL)	30 min PI	Tail vein injection
Li	2018	SD rats	Male	250–300 g	T10	Chloral hydrate (400 mg/kg）	Compression,35 g × 60 s	BMSC, miR-133b transfected BMSC	100 μg	24 h PI	Tail vein injection
Liu	2018	SD rats	Female	170–220 g	T10	Chloral hydrate (350 mg/kg)	Strike, 10 g × 12.5 mm	BMSC	200 μg	Immediately	Tail vein injection
Tsai	2018	SD rats	Female	NA	T9	NA	Strike, 10 g × 5 mm	BMSC	NA	1, 2, and 3 DPI	Tail vein injection
Wang-1	2018	SD rats	NA	200–250 g	T10	4% isoflurane and 2% isoflurane	Strike, 200 kilodyne	BMSC	200 μL (200 μg/mL)	30 min PI	Tail vein injection
Wang-2	2018	SD rats	Male	200–250 g	T10	4% isoflurane and 2% isoflurane	Strike, 200 kilodyne	BMSC	100 μL (200 μg/mL)	2 h PI (every other day subsequently)	Tail vein injection
Xu	2018	SD rats	NA	180–220 g	T9/T10	10% chloral hydrate (0.33 mL/kg)	Strike, 10 g × 25 mm	Undifferentiated PC12 cell, differentiated PC12 cell	NA	NA	Tail vein injection
Ji	2019	SD rats	Male	150–180 g	T10	60 mg/kg sodium pentobarbital	Compression, 35 g × 60 s	BMSC	100 mg	24 h PI	Tail vein injection
Guo	2019	SD rats	Male	200–250 g	T10	1–2% isoflurane, ketamine (60–90 mg/kg) and xylazine (10–15 mg/kg)	Complete transection	BMSC	40 μL	2–3 h postoperation; every other 24 h for 5 days	Intrathecal injection
Huang	2019	SD rats	Male	180–220 g	T10	60 mg/kg ketamine and 6 mg/kg xylazine	Strike, 8 g × 40 mm	EF‑MSC	100 μg	Immediately	Intrathecal injection
Rong-1	2019	SD rats	Male	180–220 g	T10	50 mg/kg pentobarbital	Strike, 10 × 12.5 mm	NSC	200 µg	Immediately	Tail vein injection
Rong-2	2019	SD rats	Male	180–220 g	T10	NA	Strike, 10 g × 12.5 mm	NSC	200 μg	Immediately	Tail vein injection
Wang	2019	SD rats	NA	180–220 g	T9/T10	10% chloral hydrate (0.3 mL/kg)	Strike, 10 g × 25 mm	PTEN siRN and miR-21/miR-19a transfected PC12 cells	NA	NA	NA
Yu	2019	SD rats	Female	230–250 g	T10	1% pentobarbital (80 mg/kg)	Strike, 200 kilodyne	BMSC	200 μg/mL	1 h PI	Tail vein injection
Zhao	2019	Wistar rats	Male	200–250 g	T10	4% isoflurane, 2% isoflurane	Compression	BMSC	500 µL/min	1 h PI	Tail vein injection
Zhou	2019	Wistar rats	Male	200–250 g	T10	2% isoflurane, 0.8 % isoflurane	Transection	BMSC	100 μg	1 h PI	Tail vein injection
Li	2019	Wistar rats	Male	150–200 g	T9–T11	10% chloral hydrate (0.33 mL/kg)	Strike, 10 g × 5 cm	BMSC	200 μg	Immediately	NA
Guo	2020	SD rats	Male	220–260 g	T10	10% chloral hydrate (3 mL/kg)	Strike, 10 g × 12.5 mm	BMSC	1 μg/μL	1 h PI	Tail vein injection
Kang	2020	SD rats	Male	180–220 g	T9/T10	10% chloral hydrate (0.33 mL/kg)	Strike, 10 g × 25 mm	miR-29b transfected PC12 cells	NA	NA	Tail vein injection
Li-1	2020	SD rats	Male	NA	L2–L5	10% chloral hydrate (3 mL/kg)	Ischemia	BMSC	5 × 10^10^ particles/100 μL	NA	Tail vein injection
Li-2	2020	SD rats	Male	NA	T10	10% chloral hydrate (3 mL/kg)	Compression, 20 s	BMSC	100 μg	24 h PI	Tail vein injection
Li-3	2020	NA	NA	NA	NA	NA	NA	MSC	NA	NA	NA
Luo	2020	SD rats	Male	170–220 g	T10	1% pentobarbital sodium (40 mg/kg)	Strike, 10 g × 12.5 mm	BMSC	200 μg	Immediately	Tail vein injection
Moham med	2020	Wistar rats	Male	250–300 g	T9 and T10	80 mg/kg ketamine and 15 mg/kg xylazine	Compression, 50 g × 5 min	NSC	10 μg	NA	Intrathecal injection
**Mouse**											
Liu	2020	C57BL/6	Male	6–8 W	T10	Halothane	Contusion, 5 g × 6.5 cm	BMSC	200 μL	Immediately	Tail vein injection
Sun	2018	C57BL/6	Female	17–22 g	T11/T12	50 mg/kg pentobarbital	10 g × 6.25 mm	hUC-MSC	1 μg/mL	30 min PI	Tail vein injection
Wang	2020	C57BL/6 J	Female	18–22 g	T10	2.0% isoflurane	Strike, 5 g × 5 cm	BMDM	200 μL	30 min PI	Tail vein injection
Zhong	2020	C57BL/6	Female	25–30 g	T10	Pentobarbital sodium	Strike, 8 g × 3 cm	NSC	200 μg	30 min PI	Tail vein injection
Yuan	2019	ICR	Male	8 W	T10	1.5% isoflurane	Strike, 50 kilodyne		20 μg	3, 6, 9, and 12 DPI	Tail vein injection
Shao	2020	C57BL/6	NA	6 W	T8/T9	30 mg/kg pentobarbital sodium	Compression, depth of 0.2 mm × 20 s	SCMEC	200 μg	1 h PI	Tail vein injection

SD, Sprague–Dawley; MSC, mesenchymal stem cell; HUVEC, human umbilical venous endothelial cell; NSC, neural stem cell; EF‑MSC, epidural fat mesenchymal stem cell; NA, not available; BMSC, bone marrow-derived stem cell; ICR, institute of cancer research; SCEMC, spinal cord microvascular endothelial cell; NSC, neural stem cells; BMDM, bone marrow-derived macrophage; DPI, day post injury; PI, post injury; hUC-MSC, human unbilical cord mesenchymal stem cell; W, week; T, thoracic; h, hour; mg, milligram; kg, kilogram; mm, millimete; cm, centimeter; s, second; min, minute.

Of the studies on rat models of SCI, one trial used an ischemic model, two trials used a transection model, three trials utilized clip compression, and the remaining trials used Allen’s model or an Infinite Horizon impactor providing a force of 200 kilodynes. Of the studies on mouse models, one used an SCI model of compression, and the rest utilized Allen’s model. Male rats and female mice were the preferred rodent models of SCI. The dose of exosomes applied in these experiments ranged from 10 to 200 μg; however, it was difficult to attain dosing information, and some trials reported only the concentration of exosomes. Exosomes were mainly injected via the tail vein and subarachnoid space within 24 h. The analyzed studies used exosomes that originated from MSCs, including bone marrow MSCs, human umbilical cord MSCs, adipose-derived MSCs, human umbilical vein endothelial cells (HUVECs), and rat pheochromocytoma (PC12) cells, as well as other cell types.

### 3.3 Comparison of BBB scores between exosome-treated and control rats

**Figure 2:**
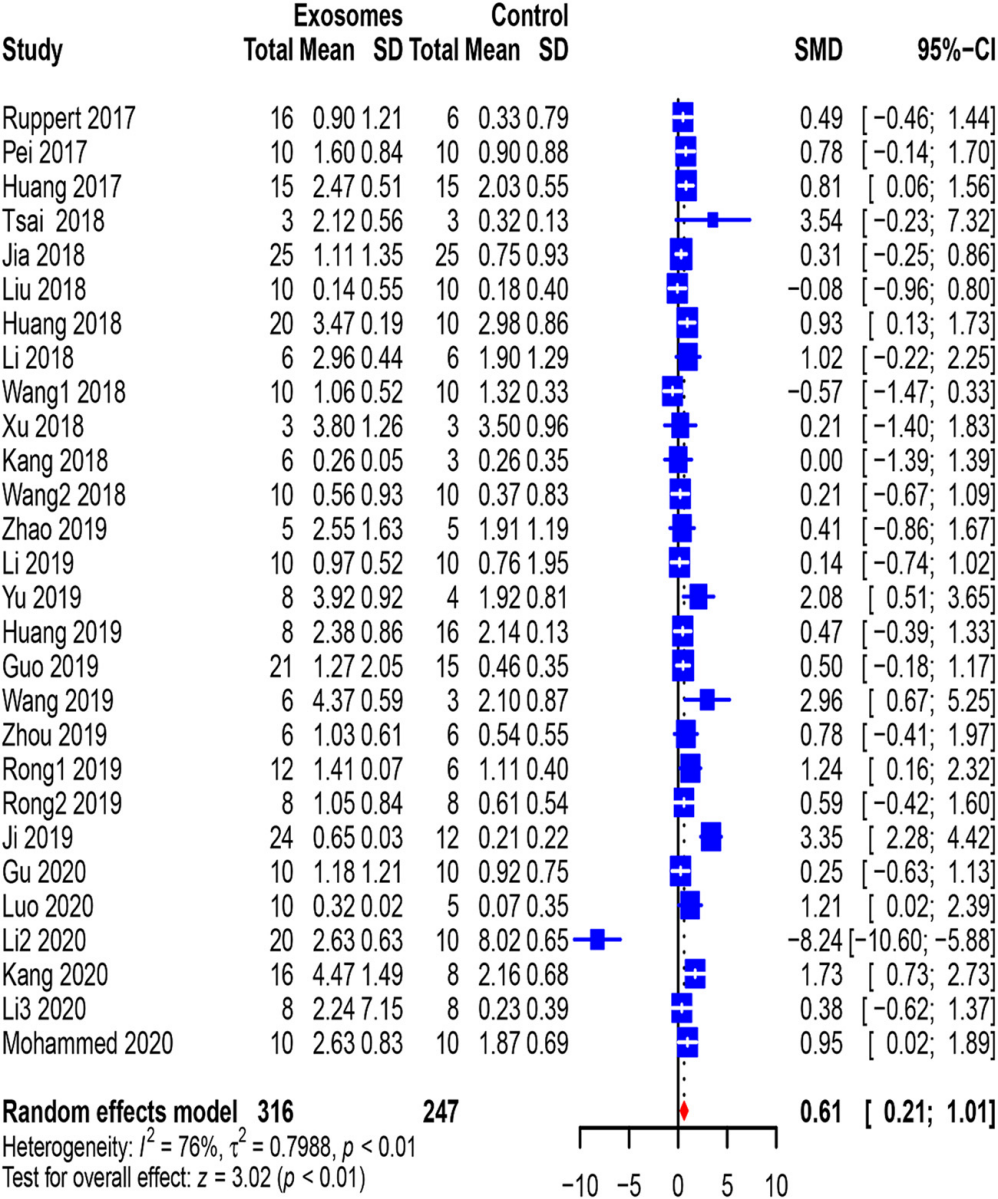
Pooled-analysis of Basso, Beattie, and Bresnahan scale at the first measurement after SCI. SCI, spinal cord injury; SMD, standard mean difference; SD, standard difference; CI, confidential interval.

**Figure 3:**
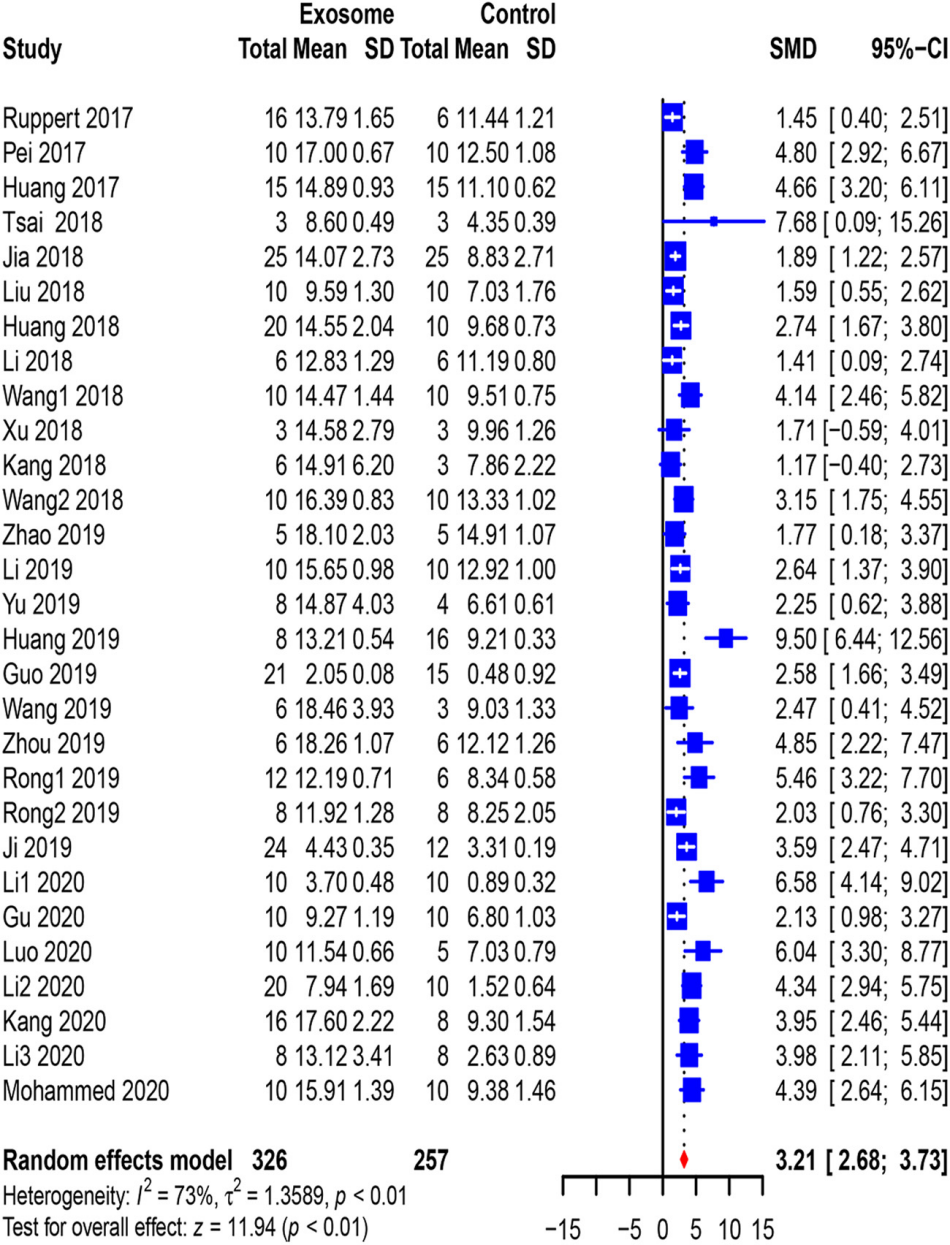
Pooled-analysis of Basso, Beattie, and Bresnahan scale at the last measurement after SCI. SMD, standard mean difference; SD, standard difference; CI, confidential interval.

We analyzed all studies (*n* = 583 animals) reporting locomotor recovery in rats at the first measurement. BBB scores reflecting the movement level of the hind limbs of exosome-treated rats were slightly but significantly improved (0.61, 95% CI: 0.21–1.01, *p* < 0.01) compared to those of rats in the control group at the first measurement (Figure 2). Furthermore, the data collected from the last measurement (3.21, 955 CI: 2.68–3.73, *p* < 0.01), which were reported in 29 studies, and the pooled analysis showed a similar outcome (Figure 3).

### 3.4 Comparison of BMS scores between exosome-treated and control mice

**Figure 4:**
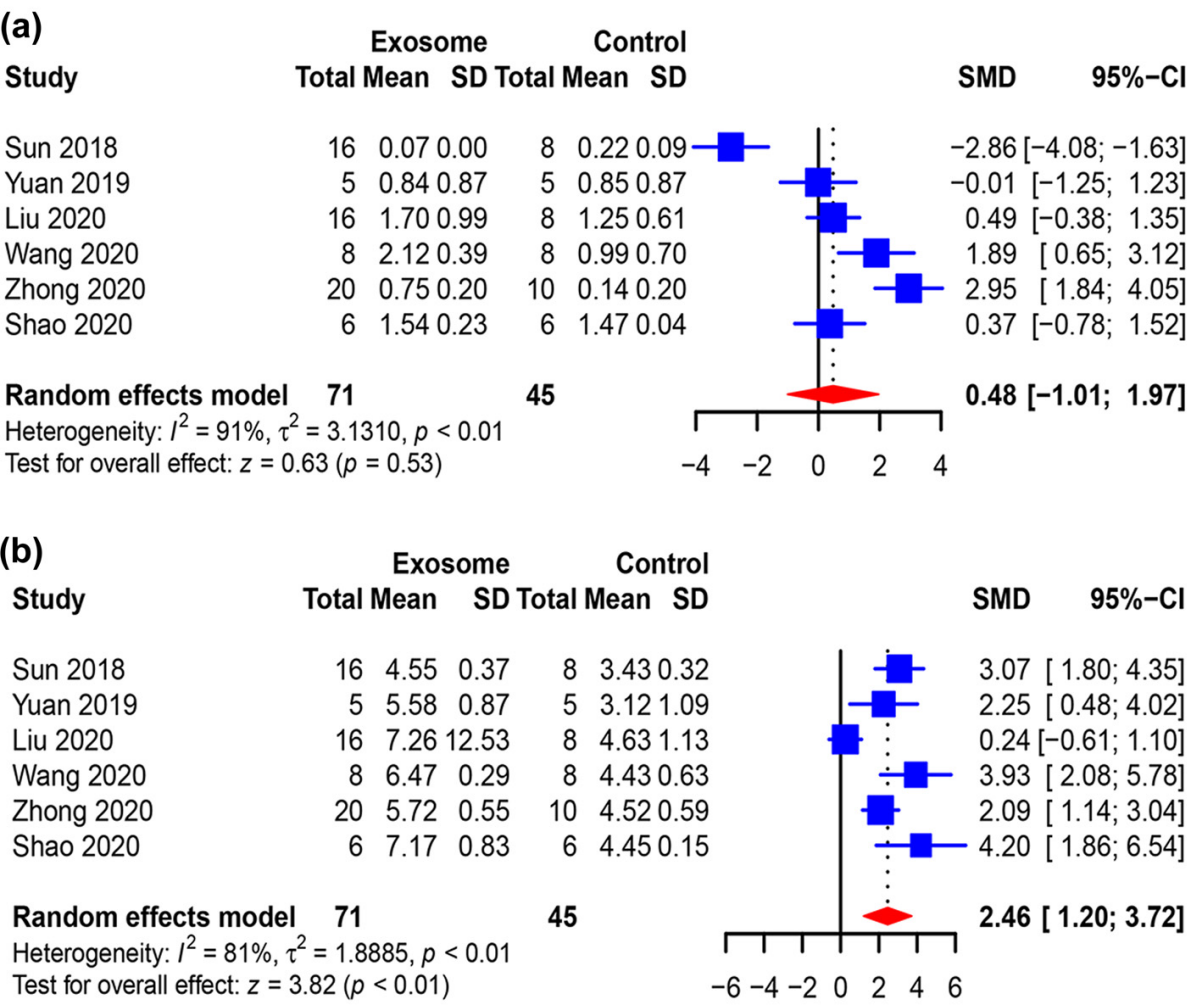
Pooled-analysis of Basso Mouse scale at the first (a) and last measurement (b) after SCI. SMD, standard mean difference; SD, standard difference; CI, confidential interval.

Six of the studies (*n* = 116 animals) evaluated the effect of exosomes on locomotor function. No remarkable improvements in the mice that received exosome administration compared to mice that received placebo administration were observed at the first measurement (0.48, 95% CI: −1.01 to 1.97, *p* < 0.01) (Figure 4a). At the last measurement, compared to placebo, exosomes increased the locomotor function of mice (2.46, 95% CI: 1.20–3.72, *p* < 0.01) (Figure 4b).

### 3.5 Trial sequential analysis

**Figure 5:**
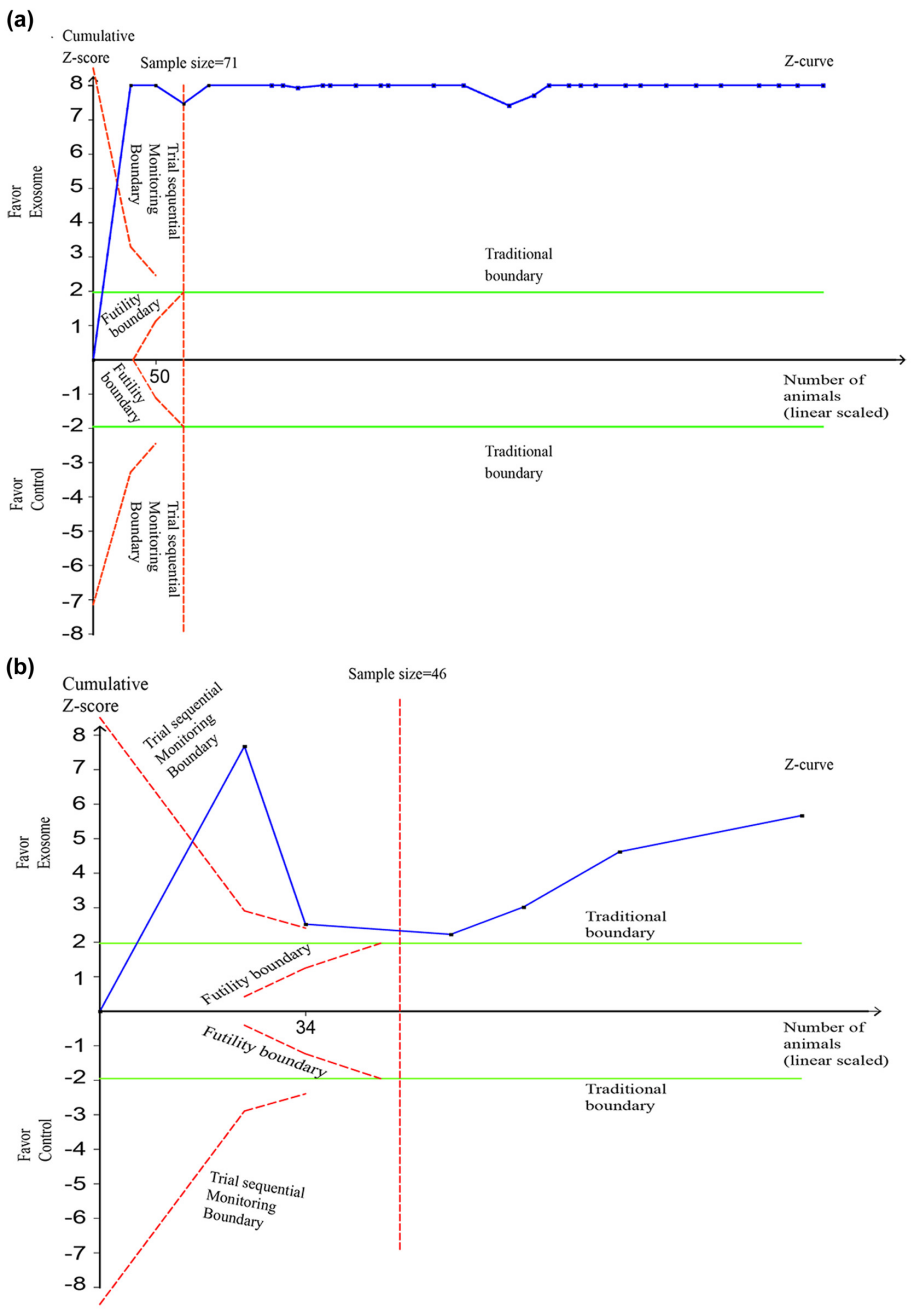
TSAs of the effect of exosomes on locomotor recovery after SCI. (a) The adjusted required information size is based on a median value of mean BBB scores of 3.21, an overall significance level (*α*) of 0.05, a type II risk (*β*) of 0.1 (power 90%), and equals 71 rats (vertical dotted red line). The cumulative *Z*-curve (solid blue line) connected by individual studies (small squares) crosses the upper O’Brien–Fleming monitoring boundary of benefit (descending dotted red line). (b) The adjusted required information size is based on a median value of mean BBB scores of 2.46, an overall significance level (*α*) of 0.05, a type II risk (*β*) of 0.1 (power 90%), and equals 46 rats (vertical dotted red line). The cumulative *Z*-curve (solid blue line) connected by individual studies (small squares) crosses the upper O’Brien–Fleming monitoring boundary of benefit (descending dotted red line).

TSAs were performed for rats and mice at the end of the follow-up day in a random-effects model meta-analysis with an overall significance level (*α*) of 0.05 and a type II error risk (*β*) of 0.1 (i.e., power 90%) preset (Figure 5). The cumulative *Z*-curve for rats crossed the upper monitoring boundary for the benefit and the adjusted required information size was calculated as 71 accrued rats, confirming a beneficial effect of exosomes on locomotor recovery (Figure 5a). Similarly, the TSA proved the beneficial effect of exosomes on locomotor recovery in SCI mice and the adjusted information size was calculated as 46 accrued mice (Figure 5b).

### 3.6 Locomotor function recovery of rats and mice on the 3rd, 7th, 14th, 21st, and 28th day post injury

**Figure 6:**
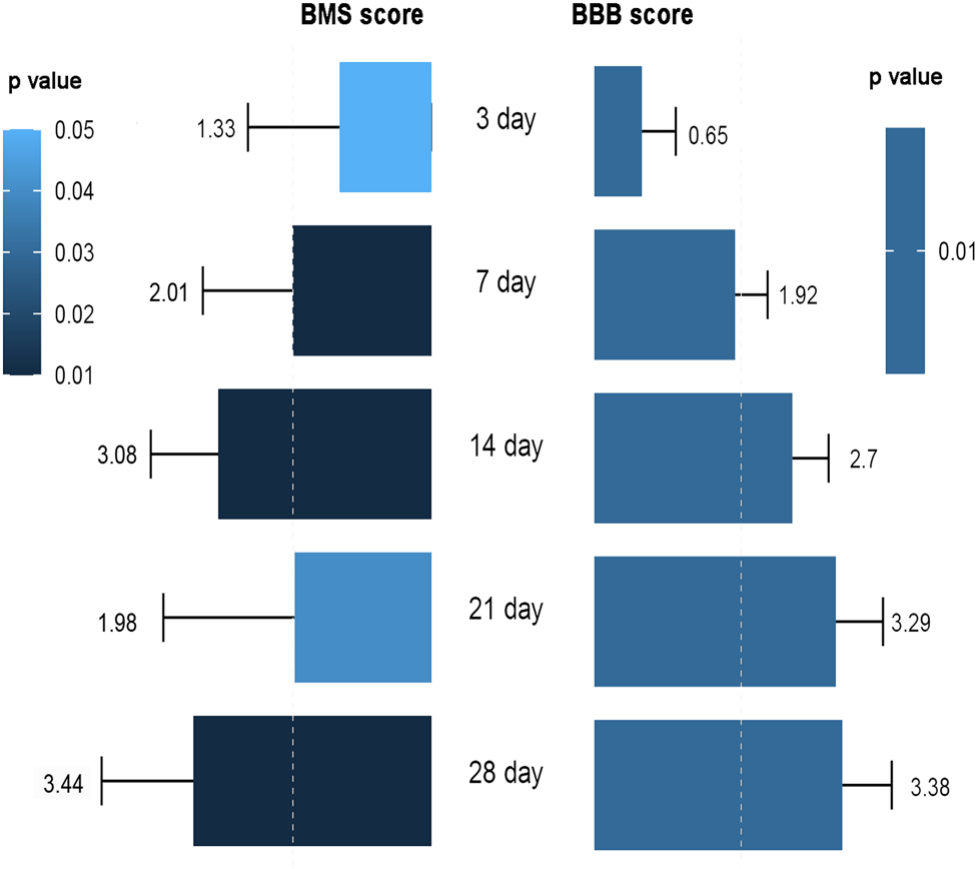
Locomotor function recovery of mice and rats on the 3rd, 7th, 14th, 21st, and 28th DPI. BBB, Basso, Beattie, and Bresnahan; BMS, Basso Mouse scale; DPI, day post injury; SMD, standard mean difference.

Most studies continuously measured the BBB scores of rats on the 3rd, 7th, 14th, 21st, and 28th day post injury (DPI; Figure 6). On the 3rd (0.65, 95% CI: 0.19–1.11, *p* < 0.01), 7th (1.92, 95% CI: 1.48–2.36, *p* < 0.01), 14th (2.70, 95% CI: 1.48–2.36, *p* < 0.01), 21st (3.29, 95% CI: 2.65–3.94, *p* < 0.01), and 28th (3.38, 95% CI: 2.71–4.05, *p* < 0.01) DPI, great improvements in locomotor function were observed in rats. Furthermore, we found that, over time, the difference prominently increased.

Meanwhile, exosome-treated mice exhibited similar improvements in locomotor function on the 3rd (1.33, 95% CI: 0.01–2.64, *p* < 0.01), 7th (2.01, 95% CI: 0.72–3.30, *p* < 0.01), 14th (3.08, 95% CI: 2.11–4.06, *p* < 0.01), 21st (1.98, 95% CI: 0.09–3.88, *p* < 0.01), and 28th (3.44, 95% CI: 2.12–4.76, *p* < 0.01) DPI. Over time, mice that received exosomes injection exhibited increasingly higher BMS scores than mice that received placebo injection (Figure 6).

### 3.7 Subgroup analysis

Four kinds of rat models of SCI (ischemia, compression, contusion, and transection) were used, and we conducted subgroup analyses of data from different rat models of SCI. The ischemic model was not subjected to subgroup analysis due to the limited number of articles that used this model (*n* = 1).

Great improvements in BBB scores were observed in contusion models (0.74, 95% CI: 0.03–1.45, *p =* 0.04), but no improvements in BBB scores were observed in the compression models (−1.25, 95% CI: −4.01 to 1.52, *p* = 0.38) at 3rd DPI; this suggested that rats in compression model trended to recover slower than rats in contusion model. On average, rats in the transection model seemed to get a higher SMD value than rats in contusion and compression models; however, this point should be cautiously concluded owing to the lack of direct evidence (Table 2).

**Table 2:** Subgroup analysis of rat models, administration modes and exosome origins

Subgroup	No. of rats (Exo)	No. of rats (SCI)	SMD	95% CI	*p* value
*SCI model*					
**Contusion**					
3d	17	17	0.74	[0.03–1.45]	0.04
7d	38	32	2.08	[0.92–3.24]	0.03
14d	38	32	3.19	[1.08–5.31]	<0.01
21d	32	26	4.79	[0.06–8.98]	0.03
28d	32	26	3.76	[1.56–5.97]	<0.01
**Compression**					
3d	41	31	−1.25	[−4.01 to 1.52]	***0.38***
7d	41	31	2.00	[0.59–3.40]	<0.01
14d	21	21	1.87	[1.10–2.64]	<0.01
21d	15	15	2.24	[−0.10 to 4.58]	***0.06***
28d	15	15	3.05	[0.50–5.61]	0.03
**Transection**					
7d	27	21	3.06	[2.17–3.95]	<0.01
14d	27	21	5.19	[3.92–6.46]	<0.01
21d	27	21	5.83	[4.43–7.23]	<0.01
28d	27	21	4.87	[3.66–6.08]	<0.01
*Administration*					
**Tail vein injection**					
3d	220	164	0.38	[−0.10 to 0.85]	***0.12***
7d	235	170	1.69	[1.17–2.21]	<0.01
14d	221	166	2.53	[1.97–3.08]	<0.01
21d	153	199	2.81	[2.14–3.47]	<0.01
28d	199	157	2.84	[2.21–3.48]	<0.01
**Intrathecal injection**					
3d	18	26	0.69	[0.06–1.32]	0.03
7d	39	41	2.22	[0.96–3.48]	<0.01
14d	39	41	4.05	[2.14–5.95]	<0.01
21d	39	41	6.26	[3.12–9.39]	<0.01
28d	39	41	5.86	[3.55–8.16]	<0.01
*Exosome origine*					
**BMSC**					
3d	149	130	0.15	[−0.48 to 0.77]	***0.65***
7d	188	154	1.58	[0.84–2.33]	<0.01
14d	188	154	3.04	[2.38–3.71]	<0.01
21d	133	124	3.09	[2.22–3.96]	<0.01
28d	168	144	3.11	[2.32–3.89]	<0.01
**Gene-modified BMSC**					
3d	12	9	0.57	[–0.36 to 1.49]	***0.23***
7d	12	9	3.11	[–1.20 to 7.42]	***0.16***
14d	12	9	1.33	[0.32–2.34]	0.01
**NSC**					
3d	30	22	0.91	[0.32–1.50]	<0.01
7d	30	22	2.31	[0.47–4.14]	<0.01
14d	30	22	2.91	[1.39–4.44]	<0.01
21d	30	22	3.68	[1.83–5.53]	<0.01
28d	30	22	3.81	[1.72–5.91]	<0.01
**PC12**					
3d	25	14	1.51	[0.20–2.81]	0.02
7d	25	14	1.26	[0.51–2.02]	<0.01
14d	25	14	0.93	[0.20–1.66]	0.01
21d	25	14	2.35	[1.43–3.26]	<0.01
28d	25	14	3.07	[2.00–4.13]	<0.01

SMD, standard mean difference; Exo, exosomes; SCI, spinal cord injury; CI, confidential intervals; PC12, pheochromocytoma; BMSC, bone marrow-derived mesenchymal stem cell.

Note: That bold italic value indicates that the SMD value between Exo and SCI group is non-significant.

Among included articles, intrathecal and tail vein injections were mainly utilized. Our subgroup analysis seemed to prefer intrathecal injection because the significant promotion of locomotor function in rats receiving tail vein injection was not observed at the 3rd DPI (0.38, 95% CI: −0.10 to 0.85, *p* = 0.12) to the 7th DPI (1.69, 95% CI: 1.17–2.21, *p* < 0.01); however, rats receiving intrathecal injection had already got significant locomotory function recovery at the 3rd DPI (0.69, 95% CI:0.06–1.32, *p* = 0.03) (Table 2).

Subsequently, we analyzed the effect of exosomes from bone marrow-derived mesenchymal stem cell (BMSC), gene-modified BMSC, neuronal stem cell (NSC), and PC12 cells. All exosomes showed satisfying therapeutic effects on SCI. However, exosomes from NSC (3rd DPI, 0.91, 95% CI: 0.32–1.50, *p < 0.01*) and PC12 cells (3rd DPI, 1.51, 95% CI: 0.20–2.81, *p < 0.01*) seemed to take effect earlier than exosomes from BMSC (3rd DPI, 0.15, 95% CI: −0.48 to 0.77, *p* = 0.65) and gene-modified BMSC (3rd DPI, 0.57, 95% CI: −0.36 to 1.49, *p* = 0.23) (Table 2). Finally, we also determined that species, year, gender, and injured segment of the spinal cord were not sources of heterogeneity by using meta-regression.

### 3.8 Bias risk

**Figure S1:**
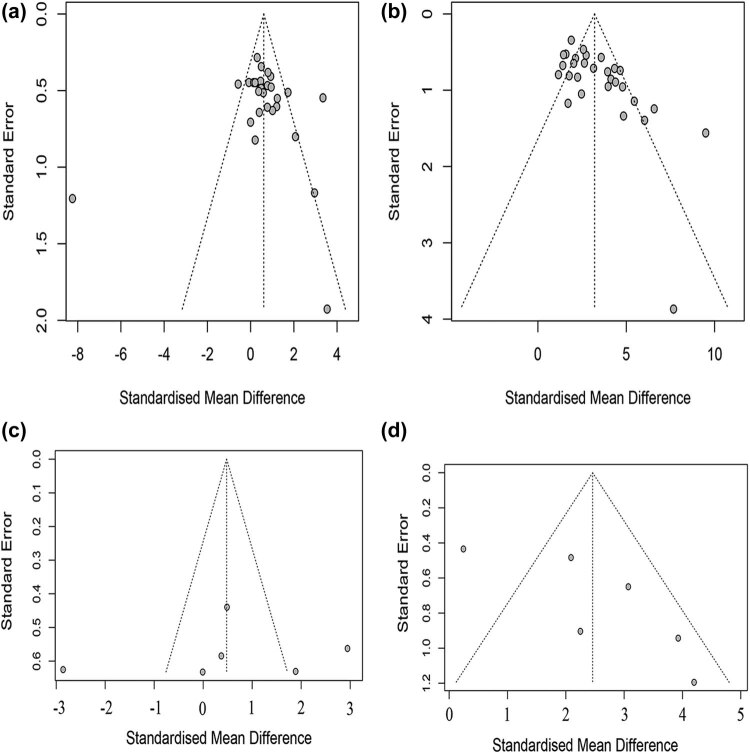
Funnel plots were conducted for pooled analysis of BBB at the first measurement (a), pooled analysis of BBB at the last measurement (b), pooled analysis of BMS at the first measurement (c), and pooled-analysis of BMS the first measurement (d).

We evaluated the article quality using SYRCLE’s tool (Table 3). The results showed that most articles reported randomness and blindness, and the rest articles reported either randomness or blindness. Other bias indexes were low. Publication biases for BBB scores at the first measurement (Figure S1a; Egger’s test, *p* = 0.907), BBB scores at the last measurement ((Figure S1b; Egger’s test, *p* = 0.00), BMS scores at the first measurement ((Figure S1c; Egger’s test, *p* = 0.767) and BMS scores at the last measurement (Figure S1d; Egger’s test, *p* = 0.066) were tested by funnel plots and Egger’s linear regression.

**Table 3:** Article quality assessment using SYRCLE’s tool

Author/Year	Random sequence	Allocation concealment	Baseline characteristics	Blinding (Study team)	Random housing	Random outcome assessment	Blinding (Outcome assessors)	Incomplete outcome data	Selective outcome reporting	
	Selection bias			Detection bias		Reporting bias		Attrition bias	Reporting bias	Other bias
**Rat**										
Huang/2017	+	?	+	−	+	−	−	+	+	+
Pei/2017	+	?	−	?	?	?	?	+	+	+
Ruppert/2017	+	?	+	?	+	+	+	+	+	+
Kang/2018	+	?	+	?	+	?	−	+	+	+
Huang/2018	+	?	−	+	+	?	+	+	+	+
Jia/2018	+	−	+	?	+	?	+	+	+	+
Li/2018	+	−	+	?	+	?	+	+	+	+
Liu/2018	+	+	+	+	+	?	+	+	+	+
Tsai/2018	−	?	+	?	−	−	+	+	+	+
Wang-1/2018	+	−	+	+	+	?	+	+	+	+
Wang-2/2018	+	?	?	−	+	−	−	+	+	+
Xu/2018	+	?	+	−	+	−	?	+	+	+
Ji/2019	−	−	+	−	−	−	?	+	+	+
Guo/2019	−	?	+	+	−	?	?	+	+	+
Huang/2019	+	?	+	+	+	?	?	+	+	+
Rong-1/2019	+	?	+	+	+	?	+	+	+	+
Rong-2/2019	+	?	+	+	+	?	+	+	+	+
Wang/2019	+	−	?	?	+	−	+	+	+	+
Yu/2019	+	−	+	?	+	−	?	+	+	+
Zhao/2019	+	−	+	+	+	?	?	+	+	+
Zhou/2019	+	?	+	+	+	?	+	+	+	+
Li/2019	+	?	+	+	+	?	?	+	+	+
Guo/2020	+	?	+	?	?	−	?	+	+	+
Kang/2020	+	?	+	?	?	−	−	+	+	+
Li-1/2020	?	?	+	?	−	−	?	+	+	+
Li-2/2020	+	?	+	+	+	?	+	+	+	+
Li-3/2020	+	?	?	−	+	?	−	+	+	+
Luo/2020	−	?	+	+	?	+	+	+	+	+
Mohammed/2020	+	?	+	−	+	?	?	+	+	+
**Mouse**										
Wei/2020	?	+	+	+	+	−	+	+	+	+
Sun/2018	?	?	+	+	+	?	+	+	+	+
Wang/2020	?	?	+	−	?	?	?	+	+	+
Zhong/2020	?	?	+	−	?	?	?	+	+	+
Shao/2020	?	?	+	+	?	+	+	+	+	+
Yuan/2019	?	?	+	+	+	?	+	+	+	+

(+) low risk of bias; (−) high risk of bias; (?) unclear risk of bias.

## 4 Discussion

To ensure reproducibility from the laboratory to the clinic, stringent animal studies should be performed, and the molecular mechanisms involved in neuroprotection should be identified. Herein, we conducted a meta-analysis of all accessible articles to assess the potential clinical translation of exosomes.

### 4.1 Summary of the evidence

This meta-analysis included 35 articles involving 699 rodents (rat, *n* = 583; mouse, *n* = 116) and compared the effects of exosomes with those of placebo. Differences of pooled analysis in the recovery of motor function of rats and mice were identified. Subgroup analysis revealed that the differences between exosome- and placebo-treated animals became greater over time. Rats in the compression model trended to recover more slowly than rats in contusion and transection models. Moreover, rats treated by intrathecal injection seemed to recover faster than rats treated by tail vein injection; however, this conclusion needs to be verified by more studies due to the lack of direct comparison. Many previous studies have reported distinct promotion of locomotor function recovery on the 7th DPI, but our findings seem to report earlier recovery on the 3rd day in rats, which is promising. Furthermore, because different rating scales were used, we should be cautious in concluding that rats recover from SCI more quickly than mice; this point should be addressed in future studies.

Rating scale (e.g., BBB and BMS) is a relatively subjective tool, especially while the score is recorded by different performers. We recommend more objective tools, such as the force of the hind limbs, motor-evoked potential (MEP), and sensory-evoked potential (SEP) while evaluating the locomotor function. Additionally, as for the experimental model for SCI, the researchers have not reached a consensus. The establishment of a standardized and globally accepted SCI model should be on the way.

As evidenced by our results, the administration method merely impacts the onset time rather than the final therapeutic effect. Thus, the tail vein injection that potentially averts secondary damage to the spinal cord is more recommended.

### 4.2 Strengths and weaknesses

To the best of our knowledge, we are the first to perform a quantitative meta-analysis assessing the curative effect of exosomes on locomotor function recovery. We carefully considered the potential origins of heterogeneity encountered in future trials, such as dose, the timing of administration and administration method, which may contribute to future clinical translation.

Limitations of this study should be addressed. We found that most studies reported positive results; hence, we hypothesized that negative results were concealed and unpublished, resulting in potential bias and misleading results. As animal trials differ from randomized clinical trials (RCTs), it is difficult to collect the characteristics of each group in animal trials, and some critical data (SCI, model dose, and administration method) were missing from these original articles. Additionally, confusing information was sometimes reported; for example, some studies provided only the volume or concentration of exosomes, and four articles did not report the injured segment of the spinal cord. Owing to the small sample size, we should be cautious to conclude the locomotor function recovery in mice. Finally, the interpretation of observations depends heavily on the individual observer and whether the observer is blinded to the treatment group. Therefore, the efficacious translation of our results should be cautious.

### 4.3 Possible mechanism of exosomes

Trauma at the lesion site directly leads to apoptosis of neurons [16], activation of cells that support neurons [11] and subsequent activation of neurotoxic signaling cascades [17] in neuronal cells. Secondary damage (mainly inflammation) triggered by microglia, astrocytes, and other immune cells, cell death, and scar formation usually occur minutes to months after SCI [18]. Currently, it is gradually acknowledged that the promotion of neuron regeneration [19], inhibition of glial activation [20], and suppression of cell death by exosomes are closely intimately with the locomotor function recovery [11]. But the steps toward inner mechanisms should never cease.

### 4.4 Implications for future studies

Animal studies are important for translation to clinical trials and evaluation of interventions for clinical trials. Identification of phenotypes, which is an important step in drug development and research, is always first performed in animals, and the mechanisms of action are later identified. Despite the large amount of evidence proving that exosomes improve the locomotor function of SCI rats [21,22,23,24], many studies have only reported that exosome administration inhibits inflammation [11,12], which is not sufficient to support a clinical trial. The complex nature of exosomes results from their components and origins. Thus, more studies investigating the mechanisms involved in neural outgrowth, inactivation of microglia and astrocytes, and inhibition of cell apoptosis should be implemented to identify the mechanism by which the greatest effects are exerted.

## 5 Conclusion

The present meta-analysis suggested that exosomes improve the locomotor function of rodents with SCI, although the mechanism of action remains investigated.

However, the SCI model, administration method, and origin of exosome are potential factors of the therapeutic effect. Our findings should be interpreted with caution considering the disparity between species and provide some insights into future studies rather than definitive clinical recommendations.

## Appendix

**Table S1:** Search strategy and databases

Database	Search strategy
PubMed	((exosomes)OR(extracellular vesicles)OR(nano-sized vesicles)OR(micro-vesicles))AND(spinal cord injury)
EMBASE	
Wed of science	
Medline	
Scopus	
Cochrane library	

**Table S2:** PRISMA 2009 checklist

Section/topic	#	Checklist item	Reported on page #
**TITLE**			
Title	1	Identify the report as a systematic review, meta-analysis, or both.	1
**ABSTRACT**			
Structured summary	2	Provide a structured summary including, as applicable: background; objectives; data sources; study eligibility criteria, participants, and interventions; study appraisal and synthesis methods; results; limitations; conclusions and implications of key findings; systematic review registration number.	2–3
**INTRODUCTION**			
Rationale	3	Describe the rationale for the review in the context of what is already known.	3–4
Objectives	4	Provide an explicit statement of questions being addressed with reference to participants, interventions, comparisons, outcomes, and study design (PICOS).	4
**METHODS**			
Protocol and registration	5	Indicate if a review protocol exists, if and where it can be accessed (e.g., Web address), and, if available, provide registration information including registration number.	NA
Eligibility criteria	6	Specify study characteristics (e.g., PICOS, length of follow-up) and report characteristics (e.g., years considered, language, publication status) used as criteria for eligibility, giving rationale.	5–6
Information sources	7	Describe all information sources (e.g., databases with dates of coverage, contact with study authors to identify additional studies) in the search and date last searched.	5
Search	8	Present full electronic search strategy for at least one database, including any limits used, such that it could be repeated.	5
Study selection	9	State the process for selecting studies (i.e., screening, eligibility, included in systematic review, and, if applicable, included in the meta-analysis).	5
Data collection process	10	Describe method of data extraction from reports (e.g., piloted forms, independently, in duplicate) and any processes for obtaining and confirming data from investigators.	6–7
Data items	11	List and define all variables for which data were sought (e.g., PICOS, funding sources) and any assumptions and simplifications made.	6–7
Risk of bias in individual studies	12	Describe methods used for assessing risk of bias of individual studies (including specification of whether this was done at the study or outcome level), and how this information is to be used in any data synthesis.	NA
Summary measures	13	State the principal summary measures (e.g., risk ratio, difference in means).	7–8
Synthesis of results	14	Describe the methods of handling data and combining results of studies, if done, including measures of consistency (e.g., I^2^) for each meta-analysis.	7–8
Risk of bias across studies	15	Specify any assessment of risk of bias that may affect the cumulative evidence (e.g., publication bias, selective reporting within studies).	7–8
Additional analyses	16	Describe methods of additional analyses (e.g., sensitivity or subgroup analyses, meta-regression), if done, indicating which were pre-specified.	8
**RESULTS**			
Study selection	17	Give numbers of studies screened, assessed for eligibility, and included in the review, with reasons for exclusions at each stage, ideally with a flow diagram.	8
Study characteristics	18	For each study, present characteristics for which data were extracted (e.g., study size, PICOS, follow-up period) and provide the citations.	8–9
Risk of bias within studies	19	Present data on risk of bias of each study and, if available, any outcome level assessment (see item 12).	NA
Results of individual studies	20	For all outcomes considered (benefits or harms), present, for each study: (a) simple summary data for each intervention group (b) effect estimates and confidence intervals, ideally with a forest plot.	9–13
Synthesis of results	21	Present results of each meta-analysis done, including confidence intervals and measures of consistency.	9–13
Risk of bias across studies	22	Present results of any assessment of risk of bias across studies (see Item 15).	12 to13
Additional analysis	23	Give results of additional analyses, if done (e.g., sensitivity or subgroup analyses, meta-regression [see Item 16]).	13
**DISCUSSION**			
Summary of evidence	24	Summarize the main findings including the strength of evidence for each main outcome; consider their relevance to key groups (e.g., healthcare providers, users, and policy makers).	14 to15
Limitations	25	Discuss limitations at study and outcome level (e.g., risk of bias), and at review-level (e.g., incomplete retrieval of identified research, reporting bias).	15–16
Conclusions	26	Provide a general interpretation of the results in the context of other evidence, and implications for future research.	18–19
FUNDING			
Funding	27	Describe sources of funding for the systematic review and other support (e.g., supply of data); role of funders for the systematic review.	19

From: Moher D, Liberati A, Tetzlaff J, Altman DG, The PRISMA Group (2009). Preferred Reporting Items for Systematic Reviews and Meta-Analyses: The PRISMA Statement. PLoS Med 6(7): e1000097. 10.1371/journal.pmed1000097.

## References

[1] Ter Wengel PV , Martin E , De Witt Hamer PC , Feller RE , van Oortmerssen JAE , van der Gaag NA , et al. Impact of early (<24 h) surgical decompression on neurological recovery in thoracic spinal cord injury: a meta-analysis. J Neurotrauma. 2019;36(18):2609–17. 10.1089/neu.2018.6277 30816058

[2] van den Berg ME , Castellote JM , Mahillo-Fernandez I , de Pedro-Cuesta J . Incidence of spinal cord injury worldwide: a systematic review. Neuroepidemiology. 2010;34(3):184–92. 10.1159/000279335 20130419

[3] Varma AK , Das A , Wallace GT , Barry J , Vertegel AA , Ray SK , et al. Spinal cord injury: a review of current therapy, future treatments, and basic science frontiers. Neurochem Res. 2013;38(5):895–905. 10.1007/s11064-013-0991-6 PMC4103794 23462880

[4] Jing Y , Yang D , Bai F , Zhang C , Qin C , Li D , et al. Melatonin treatment alleviates spinal cord injury-induced gut dysbiosis in mice. J Neurotrauma. 2019;36(18):2646–64. 10.1089/neu.2018.6012 30693824

[5] Bracken MB , Shepard MJ , Collins WF , Holford TR , Young W , Baskin DS , et al. A randomized, controlled trial of methylprednisolone or naloxone in the treatment of acute spinal-cord injury. Results of the second National acute spinal cord injury study. N Engl J Med. 1990;322(20):1405–11. 10.1056/NEJM199005173222001 2278545

[6] Eftekharpour E , Nagakannan P , Iqbal MA , Chen QM . Mevalonate cascade and small rho gtpase in spinal cord injury. Curr Mol Pharmacology. 2017;10(2):141–51. 10.2174/1874467209666160112123322 26758952

[7] Srinivas S , Wali AR , Pham MH . Efficacy of riluzole in the treatment of spinal cord injury: a systematic review of the literature. Neurosurg Focus. 2019;46(3):E6. 10.3171/2019.1.FOCUS18596 30835675

[8] Ylöstalo JH , Bartosh TJ , Coble K , Prockop DJ . Human mesenchymal stem/stromal cells cultured as spheroids are self-activated to produce prostaglandin E2 that directs stimulated macrophages into an anti-inflammatory phenotype. Stem Cell. 2012;30(10):2283–96. 10.1002/stem.1191 PMC3448872 22865689

[9] Urdzíková LM , Růžička J , LaBagnara M , Kárová K , Kubinová Š , Jiráková K , et al. Human mesenchymal stem cells modulate inflammatory cytokines after spinal cord injury in rat. Int J Mol Sci. 2014;15(7):11275–93. 10.3390/ijms150711275 PMC4139782 24968269

[10] Bagno L , Hatzistergos KE , Balkan W , Hare JM . Mesenchymal stem cell-based therapy for cardiovascular disease: progress and challenges. Mol Ther. 2018;26(7):1610–23. 10.1016/j.ymthe.2018.05.009 PMC6037203 29807782

[11] Liu W , Wang Y , Gong F , Rong Y , Luo Y , Tang P , et al. Exosomes derived from bone mesenchymal stem cells repair traumatic spinal cord injury by suppressing the activation of A1 neurotoxic reactive astrocytes. J Neurotrauma. 2019;36(3):469–84. 10.1089/neu.2018.5835 29848167

[12] Sun G , Li G , Li D , Huang W , Zhang R , Zhang H , et al. hucMSC derived exosomes promote functional recovery in spinal cord injury mice via attenuating inflammation. Mater Sci Eng C Mater Biol Appl. 2018;89:194–204. 10.1016/j.msec.2018.04.006 29752089

[13] Azmi AS , Bao B , Sarkar FH . Exosomes in cancer development, metastasis, and drug resistance: a comprehensive review. Cancer Meta Rev. 2013;32(3–4):623–42. 10.1007/s10555-013-9441-9 PMC3843988 23709120

[14] Tofaris GK . A critical assessment of exosomes in the pathogenesis and stratification of parkinson’s disease. J Parkinson’s Dis. 2017;7(4):569–76. 10.3233/JPD-171176 PMC5676982 28922170

[15] Liberati A , Altman DG , Tetzlaff J , Mulrow C , Gøtzsche PC , Ioannidis JP , et al. The PRISMA statement for reporting systematic reviews and meta-analyses of studies that evaluate health care interventions: explanation and elaboration. J Clin Epidemiol. 2009;62(10):e1–34. 10.1016/j.jclinepi.2009.06.006 19631507

[16] He Y , Zou X , Li X , Chen J , Jin L , Zhang F , et al. Activation of sodium channels by α-scorpion toxin, BmK NT1, produced neurotoxicity in cerebellar granule cells: an association with intracellular Ca (2+) overloading. Arch Toxicol. 2017;91(2):935–48. 10.1007/s00204-016-1755-2 27318804

[17] Rong Y , Liu W , Wang J , Fan J , Luo Y , Li L , et al. Neural stem cell-derived small extracellular vesicles attenuate apoptosis and neuroinflammation after traumatic spinal cord injury by activating autophagy. Cell Death Dis. 2019;10(5):340. 10.1038/s41419-019-1571-8 PMC6472377 31000697

[18] Kjell J , Olson L . Rat models of spinal cord injury: from pathology to potential therapies. Dis Model Mech. 2016;9(10):1125–37. 10.1242/dmm.025833 PMC5087825 27736748

[19] Qing L , Chen H , Tang J , Jia X . Exosomes and their MicroRNA Cargo: new players in peripheral nerve regeneration. Neurorehabil Neural Repair. 2018;32(9):765–76. 10.1177/1545968318798955 PMC6146407 30223738

[20] Xin H , Katakowski M , Wang F , Qian JY , Liu XS , Ali MM , et al. MicroRNA cluster miR-17-92 cluster in exosomes enhance neuroplasticity and functional recovery after stroke in rats. Stroke. 2017;48(3):747–53. 10.1161/STROKEAHA.116.015204 PMC5330787 28232590

[21] Xu G , Ao R , Zhi Z , Jia J , Yu B . miR‐21 and miR‐19b delivered by hMSC‐derived EVs regulate the apoptosis and differentiation of neurons in patients with spinal cord injury. J Cell Physiol. 2018;234(7):10205–17. 10.1002/jcp.27690 30387159

[22] Zhou X , Chu X , Yuan H , Qiu J , Zhao C , Xin D , et al. Mesenchymal stem cell derived EVs mediate neuroprotection after spinal cord injury in rats via the microRNA-21-5p/FasL gene axis. Biomed Pharma. 2019;115:108818. 10.1016/j.biopha.2019.108818 31102912

[23] Wang Z , Song Y , Han X , Qu P , Wang W . Long noncoding RNA PTENP1 affects the recovery of spinal cord injury by regulating the expression of miR‐19b and miR‐21. J Cell Physiol. 2019;235(4):3634–45. 10.1002/jcp.29253 PMC13482677 31583718

[24] Li C , Jiao G , Wu W , Wang H , Ren S , Zhang L , et al. Exosomes from bone marrow mesenchymal stem cells inhibit neuronal apoptosis and promote motor function recovery via the Wnt/β-catenin signaling pathway. Cell Transplant. 2019;28(11):1373–83. 10.1177/0963689719870999 PMC6802144 31423807

